# Structure and ancestry patterns of Ethiopians in genome-wide autosomal DNA

**DOI:** 10.1093/hmg/ddab019

**Published:** 2021-02-06

**Authors:** Garrett Hellenthal, Nancy Bird, Sam Morris

**Affiliations:** Department of Genetics, Evolution and Environment, University College London Genetics Institute (UGI), University College London, London, WC1E 6BT, UK; Department of Genetics, Evolution and Environment, University College London Genetics Institute (UGI), University College London, London, WC1E 6BT, UK; Department of Genetics, Evolution and Environment, University College London Genetics Institute (UGI), University College London, London, WC1E 6BT, UK

## Abstract

We review some of the current insights derived from the analyses of new large-scale, genome-wide autosomal variation data studies incorporating Ethiopians. Consistent with their substantial degree of cultural and linguistic diversity, genetic diversity among Ethiopians is higher than that seen across much larger geographic regions worldwide. This genetic variation is associated in part with ethnic identity, geography and linguistic classification. Numerous and varied admixture events have been inferred in Ethiopian groups, for example, involving sources related to present-day groups in West Eurasia and North Africa, with inferred dates spanning a few hundred to more than 4500 years ago. These disparate inferred ancestry patterns are correlated in part with groups’ broad linguistic classifications, though with some notable exceptions. While deciphering these complex genetic signals remains challenging with available data, these studies and other projects focused on resolving competing hypotheses on the origins of specific ethnolinguistic groups demonstrate how genetic analyses can complement findings from anthropological and linguistic studies on Ethiopians.

## Introduction

**Figure 1:**
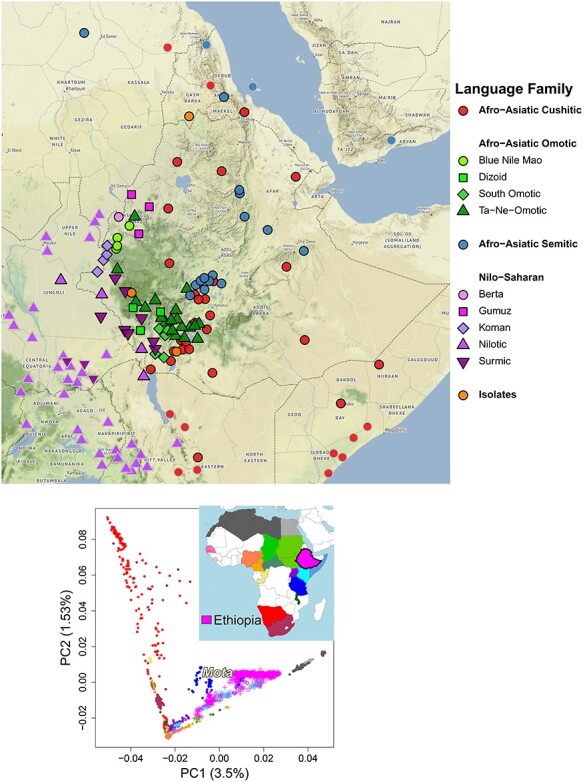
(Top) Each point on the map gives the center-point coordinates for speakers of a distinct language spoken partially/entirely in Ethiopia (black border) or only in nearby countries (gray border), using data from glottolog.org. Color/symbols denote linguistic classifications reported by glottolog.com, with these further grouped into broad categories (bold type in legend) using alternative classifications reported in ethnologue.com. (Bottom) Principal components analysis (20) of 2110 present-day Africans from 23 countries, with individuals colored by location (top right map). Symbols depict individuals from Ethiopia, Kenya, Somalia, the Sudans and Tanzania belonging to Afroasiatic- (open circles) or Nilo-Saharan (‘+’)-speaking groups, based on ethnologue.com classifications, with other individuals depicted by closed circles. ‘Mota’, a ≈4500-year-old Ethiopian described in (17), is projected onto this PCA. The proportion of variance explained by each component is given in parentheses on each axis.

Ethiopia is one of the most linguistically and culturally diverse countries in the world, composed of ethnic groups speaking over 70 different languages and residing across a wide range of topographies (Fig. 1, top). Some of the earliest hominid remains have been found in Ethiopia (1). This includes the most famous *Australopithecus afarensis* specimen named Lucy, with 40% of her osteological remains recovered (2), and Selam from 4.3 million years ago (ya), the oldest and most complete hominid (60%) to date (3–5). In addition, some of the oldest anatomically modern humans have been found in Ethiopia (6), including Omo I, dated to 190–200 kya (1,7), and the Herto fossils, dated to 154–160 kya (8). Millennia later, Ethiopia may have served as a waypoint for the initial migrations out of Africa (9).

Recent studies have generated densely genotyped autosomal DNA data from over 60 Ethiopian ethnolinguistic groups and provided new insights into their ancestral origins and genetic structure. Here, we review some of these findings, focusing on the study of genome-wide autosomal single-nucleotide polymorphism (SNP) data. We provide a current snapshot of the complex Ethiopian genetic landscape, including a description of features associated with this genetic structure, signatures of admixture and the ancestral histories of various groups. We also highlight a few of the many possible future directions in genetic studies involving Ethiopians.

## Genetic Structure of Ethiopians

Genetic data from several recent studies using both whole-genome sequencing (WGS) and genotyping arrays now allow joint analyses of genome-wide SNP data from >1000 Ethiopians representing 68 ethnolinguistic groups (10–14). Here, we use Ethiopian ethnolinguistic labels used in (12). Combining these data with those containing other Africans (15–18), primarily genotyped on the Affymetrix Human Origins array (19), we used smartpca (20,21) from EIGENSOFTv7.2.0 to perform a principal components analysis of 534 915 SNPs in individuals from >130 African ethnolinguistic groups spanning 23 countries, as described in (12). Plotting the first two principal components suggests that Ethiopians are typically more genetically similar to each other than to non-Ethiopians and are more similar to groups from other east African countries than those from the rest of Africa (Fig. 1, bottom) (12,22).

**Figure 2:**
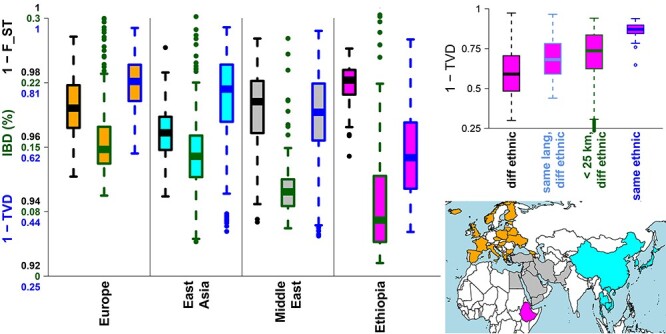
(Left) The three boxplots within each *x*-axis region show different measures of genetic similarity between all pairwise comparisons of 23 groups per geographic region, with regions defined by colors in map at bottom right: (black border) 1 − *F*_ST_ (26), (dark green border) average percentage of genome with shared IBD segments ≥2 cM (23) and (blue border) a haplotype-based genetic similarity measure based on TVD (25). Groups are defined by country (primarily in Europe) or ethnicity (otherwise). (Top right) Pairwise genetic similarity (1-TVD) among Ethiopians that have (black label) different ethnicity, (cyan) same language classification (AA Cushitic, AA Omotic, AA Semitic, NS) but different ethnicity, (dark green) location/birthplace information within 25 km of each other but different ethnicity and (blue) same ethnicity.

Consistent with their cultural and linguistic diversity, Ethiopians display a high degree of genetic heterogeneity relative to non-Africans. For example, we used hap-ibd (23), with default settings, to infer the average proportion of identity-by-descent (IBD) segments ≥2 cM shared between two people from different ethnic groups and/or countries. We also used CHROMOPAINTER (24) to infer the proportion of DNA for which each person shares most recent ancestry with 346 worldwide populations, and then we calculated the difference in these ancestry sharing proportions among people using total variation distance (TVD) (25), as described in (12) (i.e. the ‘Ethiopia-internal’ analysis described in that paper). Using either of these two haplotype-based measures of genetic similarity, on average, people from different ethnic groups in Ethiopia are less closely genetically related than people from different ethnic groups or countries in each of East Asia, Europe or the Middle East (Fig. 2).

In notable contrast to this, the median genetic distance among Ethiopian ethnic groups is lower than that seen in these other geographic regions when measured by the widely-used *F*_ST_, calculated here using HIERFSTAT (26) with default settings, that compares allele frequencies among populations (Fig. 2). A low genetic distance among some Ethiopian ethnicities when using *F*_ST_ has been reported previously (14) and may reflect how biases in the ascertainment strategies of SNP arrays can impact approaches that ignore haplotype information (27,28).

Ethiopian ethnic groups span two of the four major language phyla spoken in Africa: Afroasiatic (AA) and Nilo-Saharan (NS) (11). Several studies have shown notable genetic differences between AA and NS speakers in Ethiopia (e.g. Fig. 1) (11,12,22,29). There is also evidence of more subtle—yet significant—genetic differences among sub-categories within the AA and NS classifications, such as between AA speaking Cushitic, Omotic and Semitic groups (12,29). In general, genetic similarity among Ethiopians is notably associated with each of ethnicity, language and geography after accounting for each other (Fig. 2) (12). Researchers have also reported evidence of associations among ethnic groups’ genetic patterns and shared subsistence strategy (22) and the shared reporting of cultural practices (12).

Mapping and contextualizing this complicated genetic architecture is essential for the efficient design of genotype–phenotype association studies in Ethiopians. For example, imputing missing SNP genotypes in Ethiopians was notably improved when including the WGS data of individuals from multiple Ethiopian ethnic groups into the imputation reference panel (14). These Ethiopian WGS data (14) have already been used to help create a new Afrocentric array containing >2.2 m SNPs targeted to represent genetic diversity in multiple African populations (30). Future work will demonstrate the extent to which this array and currently available sequencing data can capture the relatively high haplotype variability across Ethiopia.

## Ancestral History of Ethiopians

While the ancestral history of Ethiopians is complex, likely in part reflecting their geographic proximity to West Eurasia, recent studies have shed light on some features of this history. Consistent with their notable genetic differences, ethnic groups speaking AA languages typically differ in their ancestry patterns to those speaking NS languages (29). For example, multiple AA speaking Ethiopian groups show evidence of admixture involving a West Eurasian-like source dated to 1500–3500 years ago (12,14,29,31), though identifying the precise West Eurasian source(s) is an area of ongoing research (32). In contrast, NS-speaking groups show evidence of more recent intermixing <1200 years ago between NS-like and other African-like sources (12), with strong genetic affinities to NS-speaking groups outside of Ethiopia and little affinity to West Eurasian sources (11,12,31). Recent work using WGS data inferred that NS and AA lineages may have diverged ≈11–16 kya (11), though it is unclear the extent to which (e.g.) differential recent West Eurasian admixture among AA relative to NS speakers may affect this inference.

The first autosomal ancient (aDNA) genome published from an African was that of a ≈4500-year-old individual found in Mota Cave in the Gamo highlands of southwest Ethiopia (17). This ‘Mota’ individual shows increased genetic similarity to particular Ethiopian ethnic groups, such as the AA Cushitic-speaking Ari (17,33), and on average, to groups living geographically nearer to where the person was found (12). Using Mota and other aDNA from Kenya and Tanzania, Prendergast *et al.* (34) infer that some east African AA-speaking groups, potentially including Ethiopian groups, descend from a mixture occurring ≈4 kya between a Mota-like source and a group previously mixed between sources related to present-day North Africans/Levantines and the NS-speaking Dinka from Sudan. Using ≈160–650 year old samples from the Pastoral Iron Age, they further infer that this (at least) three-way admixed group subsequently intermixed with a group similar to Dinka ≈2200 years ago and that some NS speakers descend from this (at least) four-way admixed population (34). They note that the different histories of NS and AA speaking groups may reflect the distinct migrations of herders into the region, as has been hypothesized by archeologists and linguists (34).

**Figure 3:**
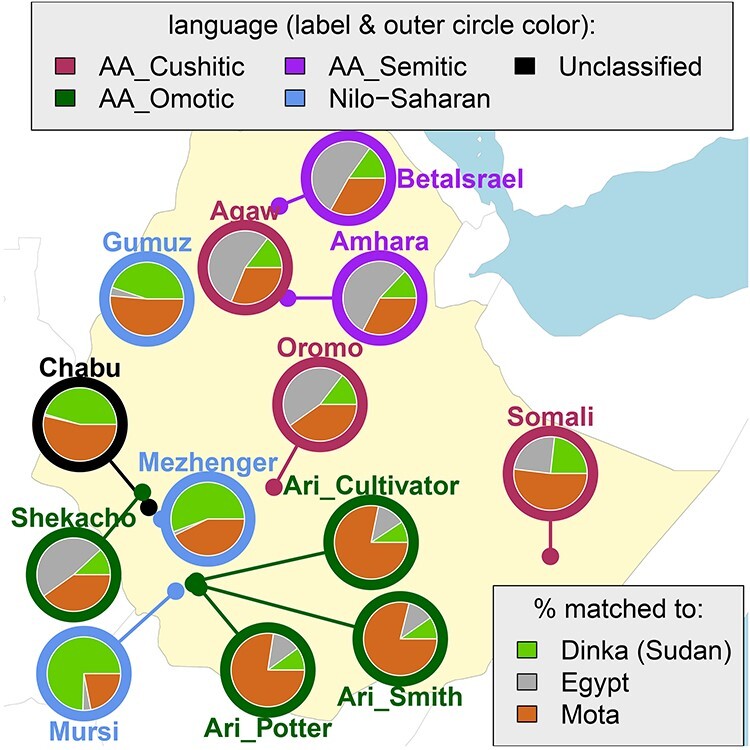
Proportion of ancestry that each Ethiopian group is inferred (using the haplotype-based models CHROMOPAINTER/SOURCEFIND) (24,35) to be most recently related to the three populations at bottom right, when using only these three populations as ancestry surrogates to mimic Figure 3 of (34). Pies are placed based on the average location information of sampled individuals in (12).

These complex, disparate admixture histories can at least partially explain the differences in ancestry patterns observed among Ethiopian ethnic groups. As an illustration, we used CHROMOPAINTER (24) and SOURCEFIND (35) to infer the relative proportions of haplotype patterns that each Ethiopian ethnolinguistic group shares with Mota and present-day Egyptians and Sudanese (Fig. 3), following the procedure described in (12), though here comparing Ethiopians to only four surrogate groups: Dinka, Mota and two Egyptian groups from (16). This relatively simple analysis, designed to mimic the surrogates used in (34), highlights some of the patterns outlined before, such as a stronger affinity to Dinka among NS speakers, more Egyptian-like DNA in AA Semitic speakers and relatively more Mota-like DNA in the Ari (Fig. 3). Broadly similar patterns have been observed when comparing Ethiopian groups to other reference populations and/or using different techniques (e.g. 11,12).

Despite these insights, several questions remain that may become clearer with new computational advances and additional data, with aDNA from relevant regions and time periods perhaps being particularly helpful. For example, while Prendergast *et al.* (34) suggest that intermixing prior to the Iron Age in the ancestors of NS speakers such as the Ethiopian Mursi may have led to their excess Dinka-like ancestry, a separate study inferred even more recent intermixing ≈600 years ago in the Mursi from sources related to NS-speaking groups and Mota that may account for some of these ancestry patterns (12). Similarly, as mentioned before, AA speakers such as the Ari show evidence of admixture that is more recent, <3500 years ago, than the ≈4.6 kya admixture inferred in Prendergast *et al*. (12,29,31,36). Recent intermixing among Ethiopian groups, which some studies have inferred (12,22), could account for some of these signals and may be obscuring ancestral differences over time.

## Genetics Records for Ethnolinguistic Groups

One difficulty in characterizing a broad genetic origin story for Ethiopians is the potential heterogeneity of histories across ethnolinguistic groups. Many groups have their own unique social customs and origin stories, with the correlation between genetics and ethnic affiliation (Fig. 2) reflecting these unique histories. To this end, recent studies have combined new genome-wide autosomal DNA with anthropological and linguistic research to shed light on the origins of particular Ethiopian ethnolinguistic groups, with two such groups being the Ari and Chabu.

### The Ari

Reflecting a pattern seen throughout southern Ethiopia, communities of Ari (Aari) people that practice farming often have limited interactions with Ari who practice artisanal activities such as blacksmithing, pottery and tanning (37,38). In general, practitioners of artisanal activities like these are among the most marginalized groups in Ethiopia, with anthropologists proposing competing theories to explain the origins of these societal divisions. One hypothesis posits that the marginalized artisanal communities reflect remnants of early groups, potentially hunter-gatherers, that occupied Ethiopia prior to the arrival of farmers (39). Another model suggests that these marginalized groups share similar ancestral origins to people from the same ethnicity who practice other occupations but have relatively recently been marginalized owing to their occupation (40).

Two separate studies analyzed genome-wide autosomal data from Ari blacksmiths and Ari cultivators in part to explore these anthropological models (29,41). Both noted strong differences in the patterns of genetic variation between them when using *F*_ST_ (42,43) and the statistical clustering algorithm ADMIXTURE (44), applying the latter under an ‘unsupervised’ setting that does not fix reference populations when inferring clusters. These strong genetic differences suggested a deep split time between the two groups, which is consistent with the Ari blacksmiths descending from a remnant community.

However, a different strategy applying the haplotype-sharing technique CHROMOPAINTER (24) to the same data, but in a manner focused on mitigating the effects of recent isolation, inferred little genetic difference between the two Ari groups (12,36). Furthermore, each Ari group, as well as Ari who work as potters, exhibited very similar inferred admixture histories (e.g. see Fig. 3) that are consistent with them becoming isolated from each other more recently than 4500 years ago (12,36). Different researchers applied IBDNe (45), which uses IBD sharing to infer changes in the effective population size over time, to these data and inferred that Ari blacksmiths have experienced a sharp decline in genetic diversity in the last 50 generations (≈1500 years), while the Ari cultivators have not (33). These findings are consistent with the relatively recent increased endogamy in the Ari blacksmiths driving the genetic differences between them and Ari cultivators, which are observed using *F*_ST_ and unsupervised ADMIXTURE.

Thus the genetic evidence overall is consistent with the model of similar ancestral origins among Ari occupational groups followed by a relatively recent marginalization related to occupation, which is the hypothesis currently favored among anthropologists (37). Similar patterns have been observed when comparing people practicing different occupations among the Ethiopian Wolayta (12). In addition to shedding light onto the ancestral origins of the Ari, this case study provides an example of the challenges in interpreting the results of widely used statistical approaches (46).

### The Chabu

The Chabu are a marginalized, isolated group of hunter-gatherers with a relatively small census size, who currently inhabit the forests in the Ethiopian highlands (33). Among several mysteries regarding their origins, the Chabu’s language currently has no classification (www.ethnologue.com). While the Chabu appear genetically distinguishable from other neighboring Ethiopian groups, three separate studies using different data and applying various methods, including those mentioned in the previous section, have shown that this is in part owing to a relatively high degree of recent isolation (12,22,33). Furthermore, these three studies all report that the Chabu are most genetically similar to NS speakers, e.g. as exemplified by the relatively higher amounts of matching to Dinka in Figure 3, which is typical of Ethiopian NS groups (Gumuz, Mursi). This suggests that despite being a linguistic isolate, the Chabu are not equally distantly related to all other Ethiopian groups, perhaps pointing to where their language may derive. One of these studies further inferred a strong recent decline in the genetic diversity among the Chabu as measured by the effective population size, which is analogous to that observed in Ari blacksmiths and which may relate to their current marginalized status (33).

Another ethnolinguistic group with an unclear linguistic affiliation is the Negede-Woyto (www.ethnologue.com), who are genetically more similar to certain AA-speaking groups than to NS speakers (12). In general, comparisons between linguistic classifications and genetic similarity can help resolve controversies or uncertainties in linguistic assignments or highlight scenarios where genetic and language transmission did not co-occur. For example, the Ethiopian Agaw are classified as AA Cushitic speakers, but they have more similar inferred admixture histories to specific AA Semitic-speaking ethnic groups that reside nearby than to any of 18 other sampled AA Cushitic-speaking Ethiopian ethnic groups (12). To make better use of DNA resources representing Ethiopians, analogous studies that focus on specific ethnic groups while combining genetic, anthropological and linguistic information are desirable. An online resource displaying which ethnic groups are most genetically similar to each other before and after mitigating recent isolation effects may be helpful in such studies (12,47).

## Future Perspectives

The emergence of genome-wide autosomal genotype and WGS data from multiple Ethiopian ethnolinguistic groups has enabled a more detailed understanding of their ancestral histories, while also laying a foundation for many additional avenues of study. For example, comparisons of autosomal data to those from the sex chromosomes and mitochondrial DNA may unearth the extent to which previously detected admixture events were sex-biased. Comparing the genomes of Ethiopians and other northeast Africans to those of non-Africans may identify the routes taken during the initial migrations of modern humans out of Africa, with one such study reporting Egypt as a more likely waypoint than Ethiopia (13). Such comparisons and other statistical techniques can also shed light on the genetic loci facilitating adaptation to the many varied environments of Ethiopia. Recent findings have discovered potential signals of natural selection in multiple Ethiopian groups, for example, involving lactase persistence (22,48) and skin pigmentation (29) in AA-speaking groups, hypoxia in AA-speaking groups living at high altitudes (49,50) and cardiovascular and immune system traits in both AA- and NS-speaking groups (11,22). Finally, more pharmacogenetic studies involving Ethiopians are necessary, with for example, Ethiopian Somali shown to have allele frequencies atypical of other worldwide populations at certain drug metabolizing enzymes that may associate with adverse drug reactions (51). Such ongoing work highlights the importance of characterizing genetic variation in Ethiopian groups and how our understanding of this complicated ancestral history and its ramifications on treatment and health today is only beginning.

*Conflict of Interest statement*. None declared.

## Funding

Sir Henry Dale Fellowship jointly funded by the Wellcome Trust and the Royal Society (098386/Z/12/Z to G.H.); the National Institute for Health Research University College London Hospitals Biomedical Research Centre; the Natural Environment Research Council (NE/L002485/1 to N.B.).
